# 2319. Analysis of the potential impact of effectiveness and availability of mRNA influenza vaccine on hospitalization and mortality

**DOI:** 10.1093/ofid/ofac492.150

**Published:** 2022-12-15

**Authors:** Fardad Haghpanah, Alisa Hamilton, Eili Y Klein

**Affiliations:** The Center for Disease Dynamics, Economics & Policy, Towson, Maryland; Center for Disease Dynamics, Economics & Policy, Washington, District of Columbia; Center for Disease Dynamics, Economics & Policy, Washington, District of Columbia

## Abstract

**Background:**

Influenza viruses constantly change because of antigenic drift. Due to the time currently needed to develop and distribute flu shots, vaccines are often ill-matched to circulating influenza strains. One silver lining of the COVID-19 pandemic was the acceleration of mRNA technology, which could significantly reduce the timeline between strain choice and deployment, potentially increasing vaccine efficacy. Significant private and public investments would be required to accommodate accelerated vaccine development and approval. Hence, it is important to understand the potential impact of mRNA technology on influenza hospitalizations and mortality.

**Methods:**

We developed a compartmental model stratified by age group to evaluate the potential effect of increased vaccine effectiveness (defined as a two-level measure of protection against infection and hospitalization) on influenza hospitalizations and mortality in the United States. We assume that mRNA technology can only shorten the time from strain choice to distribution but not distribution and administration. Thus, later decisions on vaccine composition would increase effectiveness but reduce availability. To assess this tradeoff, we evaluated two scenarios where strain choice was delayed until summer resulting in a more effective vaccine: (1) available to all age groups in the fall, or (2) available by August but only for adults 65 years and older.

**Results:**

Assuming current vaccine coverage rates, if not available until October, the vaccine would need a minimum of 80% effectiveness against infection to see a decrease in hospitalizations and deaths (Figures 1A and 1B). When delayed until November, even a 100% effective vaccine could not reduce hospitalizations or deaths (Figures 1C and 1D). For the elderly, a 50% effective vaccine against infection (Figures 1E and 1F) or a vaccine 40% effective against infection and 60% against hospitalization available in late summer was similar to an 80% effective vaccine available in October for all ages.

Age-stratified weekly number of influenza-associated hospitalization per 100,000 population and total number of deaths in the United States for an mRNA vaccine that would be available in either October (A and B), November (C and D), or by late summer but only for the 65+ age group (E and F). The Baseline represents the 10-year average weekly hospitalization rate and mortality during the Flu Season (October to May).

**Conclusion:**

As the majority of influenza-associated hospitalizations and deaths are in adults 65 years and older, a combination policy targeting higher vaccine effectiveness for this age group in the short term would be the most efficacious.

**Disclosures:**

**All Authors**: No reported disclosures.

